# Lipoxygenase Inhibition Activity of Coumarin Derivatives—QSAR and Molecular Docking Study

**DOI:** 10.3390/ph13070154

**Published:** 2020-07-17

**Authors:** Melita Lončarić, Ivica Strelec, Valentina Pavić, Domagoj Šubarić, Vesna Rastija, Maja Molnar

**Affiliations:** 1 Faculty of Food Faculty Osijek, Josip Juraj Strossmayer University, 31000 Osijek, Croatia; melita.loncaric@ptfos.hr (M.L.); ivica.strelec@ptfos.hr (I.S.); 2 Department of Biology, Josip Juraj Strossmayer University of Osijek, 31000 Osijek, Croatia; vpavic@biologija.unios.hr; 3 Department of Agroecology and Environmental Protection, Faculty of Agrobiotechnical Sciences Osijek, Josip Juraj Strossmayer University of Osijek, 31000 Osijek, Croatia; dsubaric@fazos.hr (D.Š.); vrastija@fazos.hr (V.R.)

**Keywords:** lipoxygenase, coumarins, lipid peroxidation, antioxidant activity, QSAR, molecular docking

## Abstract

Lipoxygenases (LOXs) are a family of enzymes found in plants, mammals, and microorganisms. In animals and plants, the enzyme has the capability for the peroxidation of unsaturated fatty acids. Although LOXs participate in the plant defense system, the enzyme’s metabolites can have numerous negative effects on human health. Therefore, many types of research are searching for compounds that can inhibit LOXs. The best quantitative structure–activity relationship (QSAR) model was obtained using a Genetic Algorithm (GA). Molecular docking was performed with iGEMDOCK. The inhibition of lipoxygenase was in the range of 7.1 to 96.6%, and the inhibition of lipid peroxidation was 7.0–91.0%. Among the synthesized compounds, the strongest inhibitor of soybean LOX-3 (96.6%) was found to be 3-benzoyl-7-(benzyloxy)-2*H*-chromen-2-one. A lipid peroxidation inhibition of 91.0% was achieved with ethyl 7-methoxy-2-oxo-2*H*-chromene-3-carboxylate. The docking scores for the soybean LOX-3 and human 5-LOX also indicated that this compound has the best affinity for these LOX enzymes. The best multiple linear QSAR model contains the atom-centered fragment descriptors *C-06*, *RDF035p*, and *HATS8p*. QSAR and molecular docking studies elucidated the structural features important for the enhanced inhibitory activity of the most active compounds, such as the presence of the benzoyl ring at the 3-position of coumarin’s core. Compounds with benzoyl substituents are promising candidates as potent lipoxygenase inhibitors.

## 1. Introduction

Lipoxygenases (EC 1.13.11.12, linoleate: oxygen, oxidoreductases, LOXs) are non-heme, iron-containing enzymes that catalyze the deoxygenation of polyunsaturated fatty acids containing a 1-*cis*,4-*cis*-pentadiene structure, resulting in the formation of conjugated diene hydroperoxides [1,2]. LOXs are widespread in the plant and animal kingdoms but also found in microorganisms such as fungi and cyanobacteria [3]. LOXs can be found in the biological organs and tissues, but they are particularly abundant in legumes (soybeans, mung beans, green beans, navy beans, peas, and peanuts), cereals (rye, oat, wheat, corn, and barley), and potato tubers [4,5]. All biological systems are susceptible to lipid peroxidation. LOXs are very important enzymes in plants, because of many lipoxygenase pathway products such as jasmonates, leaf alcohols, and antimicrobial and antifungal compounds such as leaf aldehydes and divinyl ethers [6,7]. All these products play an important role in the plant’s interaction with pathogens, insects, or abiotic stress [8]. In the food industry, LOXs are used as bleaching agents (bread and pasta making) [9] and in aroma production [4,10]. In addition, LOXs can cause food spoilage (off-flavors and off-odors) due to their reactions with unsaturated fatty acids [11]. Lipids and molecular oxygen are involved in these reactions, and they can be accelerated by many factors (singlet oxygen, light, metal ions, and radiation), including enzymes containing a transitional metal prosthetic group such as LOXs [5]. For that reason, many types of research have been conducted in order to find LOX inhibitors. Different organic compounds are reported as LOX inhibitors such as coumarins [12,13], rhodanines [14,15], thiazolidinediones [16], and quinazolinone-1,2,3-triazoles [17].

Among the organic compounds acting as LOX inhibitors, coumarins should be pointed out as natural compounds that can be found in plant roots, leaves, flowers, seeds, fruits, and bark, but their isolation from plants is time-consuming and expensive [18,19]. They are also interesting compounds due to the possession of various medicinal properties such as anti-inflammatory, anti-HIV, anti-proliferative, anticoagulant, anti-allergic, antimicrobial, antioxidant, antidepressant, antinociceptive, antitumor, antiviral, antiasthmatic, anti-Alzheimer’s, anti-influenza, antipyretic, antihyperlipidemic, and antituberculosis effects [20,21]. Coumarins can also inhibit some other enzymes such as acetylcholinesterase, β-secretase, and monoamine oxidase [22]. Iranshahi et al. [23] reported a significant inhibition of the soybean lipoxygenase by coumarin umbelliprenin. Among 12 tested coumarins reported by Melgarki et al. [13], 11 of them were found to inhibit lipoxygenase by 8–100%. On the other hand, the coumarins reported by Kontogiorgis and Hadhipavlou-Litina [12] were found to be very ineffective lipoxygenase inhibitors (with 16.1–22.6% inhibition). 

The lipoxygenase family catalyzes diverse physiological processes in both plants and animals. Although LOXs’ biochemical characterizations have been performed mainly on the soybean LOX isoforms, LOXs can be found in mammals as well. In humans and mice, six LOX isoforms have been found. The human enzyme 5-lipoxygenase (5-LOX) initiates the biosynthesis of the proinflammatory leukotriene (LT) lipid mediators required for the stimulation of inflammatory reactions. LT production is regulated by including Ca^2+^-targeted membrane binding and phosphorylation at specific serine residues [24]. Most of the LOX inhibitors are antioxidants or free radical scavengers since lipoxygenation occurs via a carbon-centered radical. Coumarin derivatives have demonstrated effective lipoxygenase (LOX) inhibition activity [25,26]. An unsubstituted phenyl ring and compounds with a 2-Me group on the phenyl ring and higher lipophilicity values showed good anti-inflammatory activity [26]. In soybeans, four seed isoforms—LOX-1, LOX-2, LOX-3a, and LOX-3b—have been identified. LOX-1 and LOX-3 have two domains with similar secondary structural elements: an N-terminal domain with flexible loops and α-helix-rich C-terminal catalytic domain [27]. A comparison of the binding site of human 5-LOX with that of soybean LOX-3 has revealed that that the amino acids in the binding sites of human 5-LOX have much similarity with those in the soybean LOX-3 enzyme [26].

Many authors have reported coumarin synthesis, but most methods have included toxic solvents or catalysts [19]. The coumarins used in this research were synthesized by green methods and techniques without the use of toxic solvents. All the compounds were synthesized in a solvent-free manner or in deep eutectic solvents (DESs). 

Based on the published research and our interest in coumarin derivatives, in this paper, we present in vitro lipid peroxidation and the soybean lipoxygenase inhibitory activity of coumarins. In addition, the antioxidant activity of examined coumarins was tested. In order to signify the importance of the structural and chemical attributes for the lipoxygenase inhibition for the series of coumarin derivatives, quantitative structure–activity relationship (QSAR) analysis was performed. The binding affinity and interactions with the active sites of soybean LOX-3 and human 5-LOX were evaluated by molecular docking and compared. 

## 2. Results and Discussion

### 2.1. Synthesis of Coumarin Derivatives

The synthesis of the tested compounds was performed as described previously [28]. Five series of compounds were synthesized via Knoevenagel condensation in a reaction between substituted salicylaldehydes and active methylene compounds (ethyl acetoacetate, ethyl cyanoacetate, dimethyl malonate, diethyl malonate, and ethyl benzoylacetate). The structures of the analyzed compounds are presented in Table 1.

### 2.2. Inhibitory Activity

The synthesized coumarin derivatives were tested for inhibitory activity against soybean lipoxygenase at 100 µM concentrations in the reaction mixture, and the results are shown in Table 1. It should be mentioned that the inhibitory activities of compounds **38** and **39** were not possible to determine since the reaction mixture was cloudy, probably due to the low solubility of the tested compounds. Five series of coumarin derivatives were synthesized in the reaction of substituted salicylaldehydes and dimethyl malonate (Series 1—**2**, **12**, **16**, **20**, **27**, **30**, **36**), diethyl malonate (Series 2—**3**, **17**, **21**, **24**, **26**, **28**, **33**, **37**), ethyl benzoylacetate (Series 3—**5**, **7**, **13**, **23**, **25**, **35**, **38**, **39**), ethyl cyanoacetate (Series 4—**4**, **6**, **10**, **18**, **22**, **29**, **31**, **34**), and ethyl acetoacetate (Series 5—**1**, **9**, **11**, **14**, **15**, **19**, **32**). 

Substituted methyl 2-oxo-2*H*-chromene-3-carboxylates (Series 1) inhibited soybean LOX-3 by 7.1–85.1%. The highest inhibition rate of 85.1% was found for methyl 6-bromo-2-oxo-2*H*-chromene-3-carboxylate (**16**). For the compounds of Series 2, the highest inhibition rate of 56.1% was found for ethyl 6, 8-dibromo-2-oxo-2*H*-chromene-3-carboxylate (**26**), followed by that for ethyl 6-bromo-2-oxo-2*H*-chromene-3-carboxylate (**17**) with an inhibition activity of 55.2%. 6-bromo-2-oxo-2*H*-chromene-3-carbonitrile (**18**) acts as an efficient LOX inhibitor (84.8%) of Series 4. When comparing all the compounds with the bromine substituent (**16**, **26**, **17**, **18**), it could be concluded that the bromine contributes to the lipoxygenase inhibition. Compound **15** also had substituted bromine in its structure but did not show as much inhibition activity as the other compounds from Series 5. The highest inhibitory activity for all the compounds was observed with 3-benzoyl-7-(benzyloxy)-2*H*-chromen-2-one (**7**), from Series 3 (96.6%). Series 5 had the lowest range of inhibition (11.3–48.5%) compared to other the compound Series (1–4). It could be observed that the compounds without substituents in positions 6, 7, and 8 of the coumarin core (**1**, **2**, **3**, **4**, and **5**) showed very low inhibition. The 10 most active soybean LOX inhibitors among tested the coumarins are compounds **7**, **16**, **18**, **13**, **30**, **26**, **23**, **17**, **20**, and **21**, and all of them have a substituent in position 6 of coumarin core, except compounds from Series 3 (**7** and **13**), which have substituents in position 7 of the coumarin core. Inhibitory activity against lipoxygenase exhibited by coumarin derivatives was previously reported [29,30]. Roussaki et al. [31] examined twelve 3-aryl coumarins, and only half of them were found to be LOX inhibitors. Soybean LOX-3’s inhibition activities were in the range of 6–86%, and the highest inhibition rate of 86% was achieved with a compound with bromine in position 6 of the coumarin. Poor inhibition (16.1–22.6%) was achieved with the coumarins reported by Kontogiorgis and Hadjipavlou-Litina [12]. Only three coumarins of seven showed some inhibitory activity. The inhibitory activity of coumarin (36%) was higher than that presented in Table 1, while the value of coumarin inhibition reported by Symenidis et al. [32] was even lower (15%). Melagraki et al. [13] examined coumarin-3-aminoamides. Only one of the twelve tested coumarins was not active, and the inhibition was in the range of 8% to 10%. 

The inhibition of lipid peroxidation was also examined for coumarin derivatives, and the results are presented in Table 1. Lipid peroxidation inhibition was in the range of 7.0–91.0%. Compounds of Series 1, 2, 3, 4, and 5 showed lipid peroxidation inhibition ranges 16.6–86.6%, 7.0–91.0%, 42.1–86.1%, 12.9–81.0% and 23.0–69.0%, respectively. The compounds with the highest inhibition rates of 69.2–91.0% (**39**, **15**, **38**, **30**, **17**, **18**, **31**, **7**, **16**, and **28**) all have substituents in position 6 or 7 of coumarin. All the coumarins with bromine in position 6 (**16**, **17**, **38**, **18**, and **15**) had a high rate of inhibition except compound **26** (31.1%). In addition, four compounds with the methoxy group in position 6 or 7 of the coumarin core (**39**, **30**, **31**, and **28**) are among the compounds with the highest inhibition. 3-acetyl-6-hydroxy-2*H*-chromen-2-one (**19**), 6-hydroxy-2-oxo-2*H*-chromene-3-carbonitrile (**22**), and 3-benzoyl-6-hydroxy-2*H*-chromen-2-one (**23**) are the compounds with hydroxyl group in position 6 of the coumarin core, and showed very similar rates of inhibition: 69.2%, 66.8%, and 66.5%, respectively. Compound **4** (2-oxo-2*H*-chromene-3-carbonitrile) showed no inhibition. All the results were compared with an appropriate standard inhibitor, Trolox, that showed inhibition of 61.8%, which is in accordance with the literature. Inhibitions of 63% for Trolox were previously reported [32,33,34]. Coumarin’s (**8**) inhibition of 2.6% is negligible, and it is in accordance with reported data showing that coumarin is completely inactive [35].

The interaction of the examined coumarin derivatives with the stable free radical 1-diphenyl-picrylhydrazyl (DPPH) is shown in Table 1. The compounds showed moderate antioxidant activity in the range of 15.2 to 58.1%. Ethyl 6-hydroxy-2-oxo-2*H*-chromene-3-carboxylate (**21**) showed the highest antioxidant activity, followed by the three compounds (**18**, **10**, and **22**) with the carbonitrile group in position 3 of the coumarin core. Compounds **10**, **22**, and **23** are coumarins with very similar rates of antioxidant activity: 39.2, 39.0, and 38.9%, respectively. All three compounds possess hydroxyl groups, **22** and **23** in position 6 and compound **10** in position 8 of the coumarin. The results are compared with the appropriate standards Trolox and NDGA. 

### 2.3. QSAR Study

The best model obtained for lipoxygenase inhibition is:

log % inhibition = 0.73 + 0.40 (0.66) *C-026* + 0.06(0.34) *RDF035p* − 1.59(−0.30) *HATS8p* (1)

*N*(training) = 29; *N*(test) = 8 (**16**, **19**, **22**, **24**, **25**, **28**, **35**, **37**).

The statistical parameters of the obtained models are given in Table 2. The variables in Equation (1) are listed in order of relative importance by their standardized regression coefficients (β, in brackets). However, according to the statistical results presented in Table 1, model (1) does not satisfy the threshold for fitting and external validation parameters: *R^2^*_train_ > 0.60 and *R^2^*_test_ < 0.06. Since compound **14** from the training set has a standard residual of −2.65 (greater than 2), it has been deemed an outlier. This can be explained since it is the only compound with a 7-diethylamino group (Table 1). After the removal of compound **14** from the training set, subsequent re-analysis produced the following improved QSAR model:

log % inhibition = 0.67 + 0.42 (0.72) *C-026* + 0.07 (0.43) *RDF035p* − 1.85 (−0.36) *HATS8p* (2)

**Figure 1:**
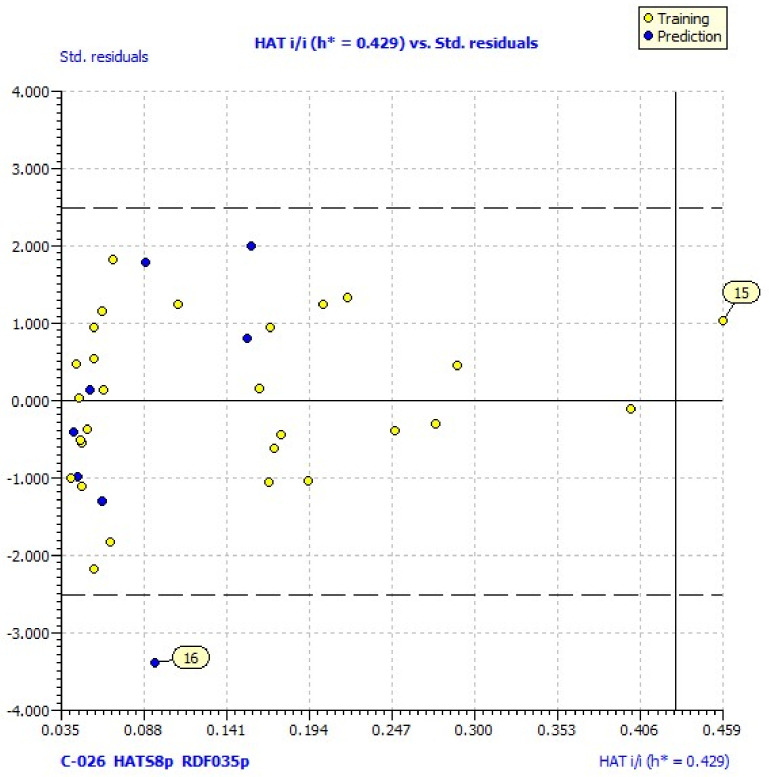
Williams plot of applicability domain of the QSAR model for lipoxygenase inhibition calculated by model (2).

The values of the descriptors included in models (1)–(2) are given in the Supplementary Materials Table S1. The values of log % inhibition calculated by Equation (2) for each molecule are presented in Table 1 and Table S1. The statistical parameters for the model (2) are presented in Table 2. In order to exclude the possibility that the models were overfitted, the collinearity of the descriptors included in the QSAR models was evaluated with a correlation matrix (Table 3). Low collinearity was confirmed by the values of the correlation coefficient (R), ≤ 0.7, and verified by the low values of Kxx and ΔK ≥ 0.05 (Table 2). The model satisfied the fitting and internal validation criteria: R^2^ and R^2^_adj_ ≥ 0.60 (the closer the R^2^ values are to unity, the more similar the calculated values are to the experimental ones); CCC_tr_ ≥ 0.85; a root-mean-square error (RMSE) and mean absolute error (MAE) close to zero; and RMSE_tr_ < RMSE_cv_ (Table 2) [36]. The cross-validated correlation coefficient (Q^2^_loo_ = 0.06) demonstrates a satisfactory internal prediction power for model (2). Y-scrambling or response permutation/randomization testing is a technique used to check the robustness of a QSAR model. The robustness of the obtained QSAR model was affirmed by R^2^_y_scr and Q^2^_y_scr values < 0.2, as R^2^_y_scr > Q^2^_y_scr [37]. Model (2) satisfied some of the external validation criteria: R^2^_ext_ ≥ 0.60, low RMSE and MAE, and low differences between RMSE_tr_ and RMSE_ex_ as well as between MAE_tr_ and MAE_ex_. However, the negative values of Q^2^_F1_, Q^2^_F2_, and Q^2^_F3_—lower than 0.6—and r^2^_m_ average lower than 0.6 indicate that this model is useless for external prediction [38]. Williams plots for the same models reveal one outlier (compound **16**) and one compound outside of the applicability domain (**15**) (Figure 1). Its laverage (HAT = 0.583) is greater than the warning laverage (h* = 0.429); therefore, the predicted value for compound **15**, which is poorly active (% LOX inh. = 11.25), must be interpreted with great care.

The largest value of the standardized regression coefficients in Equation (2) has a descriptor C-026 that belongs to the atom-centered fragments group of descriptors [39]. Since the atom-centered fragment approach decomposes the molecule into structural pieces, these kind of descriptors take into consideration the local physicochemical properties of any part of a molecule. Thus, the descriptor *C-026* represents the occurring atomic states of R-CX-R carbon, where X could be any heteroatom (O, N, or halogens). The positive coefficient of *C-026* in Equation (2) indicates that higher values of that descriptor are favorable for lipoxygenase inhibition. For comparison, the most active compound against lipoxygenase, compound **7**, has two R-CX-R carbon atoms, one from 3-benzoyl and second from a 7-benzyloxy group, and the % of LOX inhibition is 96.6%. Compound **5** has only one R-CX-R carbon atom from a 3-benzoyl group, and the % of LOX inhibition is decreased to 22.5% (Table 1). The R-CX-R group corresponds to the presence of the alkoxy group, a structural feature that decreases the hydrophobicity of molecules [34,40]. According to Viswanadhan et al. [39], the atomic hydrophobicity of the C atom in the R-CX-R group is low (log *p* = −0.103). Hydrophobicity is a property that governs the interaction of the ligand (drug) molecules with the biological receptor. Thus, the oxygen atom from the benzoyl group can act as an H-bond acceptor and could form H bonds with amino acid residues. 

**Figure 2:**
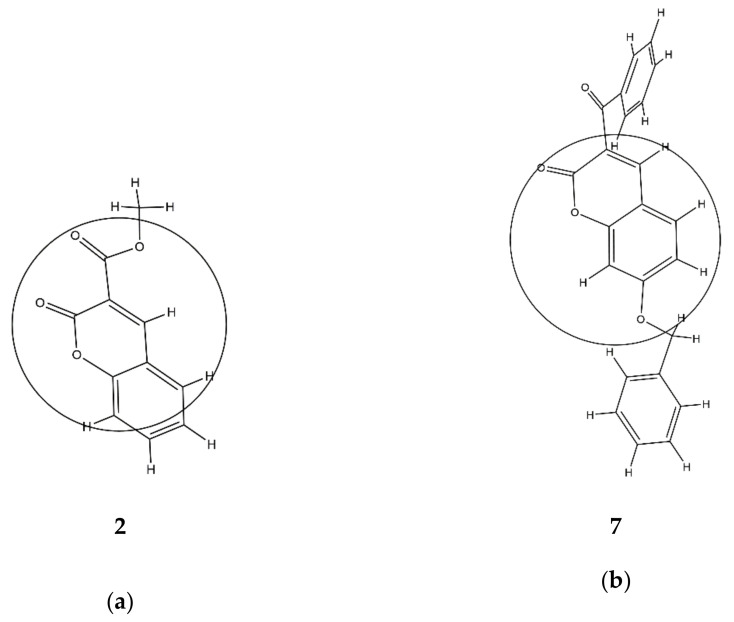
3D molecular distribution of atoms in radii of 3.5 Å from the geometrical centers of the molecules: (**a**) the least active, compound **2** (*RDF035p* = 2.59), and (**b**) the most active, compound **7** (*RDF035p* = 7.003).

The second variable in Equation (2) is a descriptor calculated by the Radial Distribution Function (RDF) approach [41]. The *RDF* of an ensemble of N atoms can be interpreted as the probability distribution for finding an atom in a spherical volume of radius *r*. The presence of the descriptor *RDF035p* in Equation (2) suggests the occurrence of some linear dependence between the inhibition power and the 3D molecular distribution of polarizabilities, calculated for the radius of 3.5 Å from the geometrical center of each molecule. Molecules with more atoms with higher polarizabilities in this area, such as carbon (*p* = 1.76) [42], have higher values of *RDF035p*. Thus, compound **7** has a higher value of *RDF035p* and therefore better inhibition ability than compound **2** since it has more carbon compounds and fewer oxygen atoms (*p* = 0.802) in an area of 3.5 Å from the geometrical center of each molecule (Table S1, Figure 2). This supports previous findings that the replacement of a phenyl group attached on the coumarin ring by a 2-pyridyl group or by a morpholinyl group decreased the inhibitory activity of 6- and 7-substituted coumarins against lipoxygenase [43]. Additionally, in the study of lipoxygenase inhibition by *O*-prenylated 3-carboxycoumarins, *O*-isopentenyl derivatives demonstrated no considerable lipoxygenase inhibition, while *O*-geranyl and *O*-farnesyl derivatives demonstrated potent inhibitory activity [44]. 

The third variable, descriptor HATS8p, is the leverage-weighted autocorrelation of lag 8/weighted by atomic polarizabilities, which belongs to the GETAWAY (GEometry, Topology, and Atom-Weights AssemblY) descriptors [42]. The values of this descriptor depend on the 3D relative atom location in the molecule. Therefore, compounds with a larger number of atoms with enhanced atomic polarizabilities, especially Cl and Br at the topological distance of 2, possess higher HATS8p values, such as **15** (0.282), which has a lower inhibitory effect.

### 2.4. Molecular Docking Study

#### 2.4.1. Molecular Docking with 5-LOX

For the present study, we have chosen the human lipoxygenase, 5-LOX, in complex with arachidonic acid (PDB ID: 3V99) [24]. The docking scores of the docked poses with total energy <100 kcal mol^−1^ are presented in Table 4. The docking scores and main interaction energies of the best docking poses of all the compounds—including the standard ligand, arachidonic acid—are presented in Supplementary file 2 (Table S2). A docking study indicated that all the tested compounds (**1–37**) exhibited relatively high potential for binding to the active site of 5-LOX. The compounds were ranked by the total energies of the predicted poses in the binding sites.

**Figure 3:**
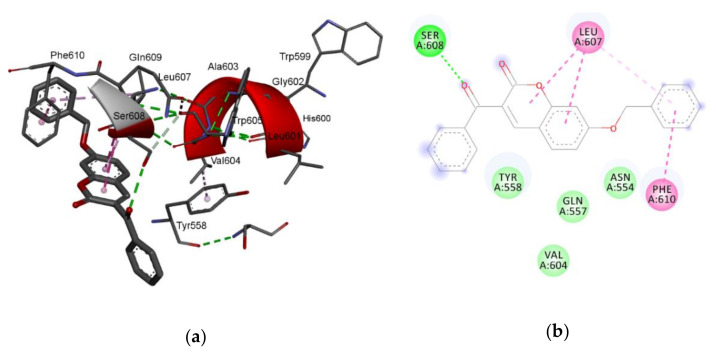
The main interactions of compound **7** with residues in the binding site of human lipoxygenase 5-LOX: (**a**) 3D representation of the binding site; (**b**) 2D representation (green = conventional hydrogen bond; light green = van der Waals; purple = *π*–σ interactions; light purple = *π*–*π* interactions).

**Figure 4:**
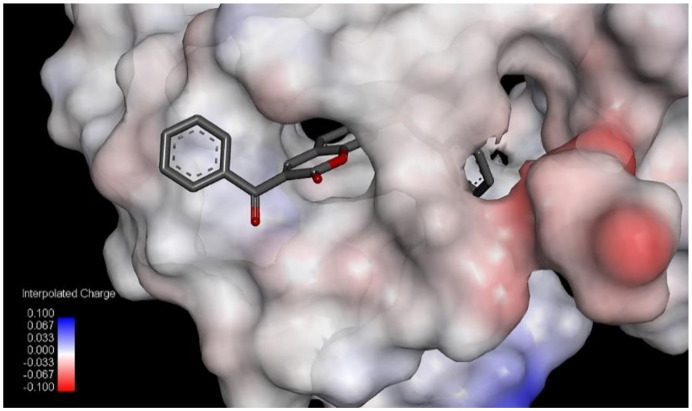
Charge surface representation of 5-LOX binding site with docked compound **7**.

As shown in Table 4, compound **7** has the lowest binding energy (−126.2 kcal/mol); therefore, it best fits into the active site of 5-lipoxygenase, even better than the standard ligand, arachidonic acid. This is consistent with the fact that compound **7** is also the most active compound (Table 1). The energies of the interactions between the protein residue and ligand **7** in docked pose 2 are tabulated in Table 5. Figure 3 illustrates the interactions of ligand **7** with the receptor 5-lipoxygenase in the binding site. A charge surface representation of the 5-LOX binding site with docked compound **7** is presented in Figure 4. The active site of 5-LOX is an elongated cavity (Figure 4), lined with invariant and 5-LOX specific polar residues that have the ability to interact with ligands during the binding process [24]. Compound **7** formed three hydrogen bonds, with ASN554, SER608, and GLN557. The oxygen atom from the benzoyl group acts as a H-bond acceptor and formed a H bond with the side chain of SER608 (2.67 Å). A strong π–π interaction is formed between the 3-benzoyl ring and side chain of PHE610 (4.34 Å). LEU607 creates two amide–π interactions with chromone rings (4.27 and 4.75 Å), and two π–alkyl interactions with the benzene ring from chromene (5.42 Å) and the 3-benzoyl ring (5.43 Å). The fact that the first three compounds, ranging in docking score energy (Table 4), have the 3-benzoyl ring as a substituent indicates the importance of that substituent for the generation of interactions. The other compounds formed interactions with the same key residues, but only a few created hydrogen bonds with SER608 (compounds **7**, **9**, and **10**) (Table S2). Molecular docking confirmed the previous findings regarding the characteristic binding interactions of coumarin derivatives with the 5-LOX binding site, which formed interactions with key amino acid residues such as HIS372, GLY557, LYS409, GLN413, HIS550, ASN554, and TYR558. A docking study of 7-substituted coumarin derivatives also revealed the importance of the same oxygen atom, which acts as a H-bond acceptor, forming a H-bond with GLY557 (2.8 Å) [26].

#### 2.4.2. Molecular Docking with Soybean LOX-3

**Figure 5:**
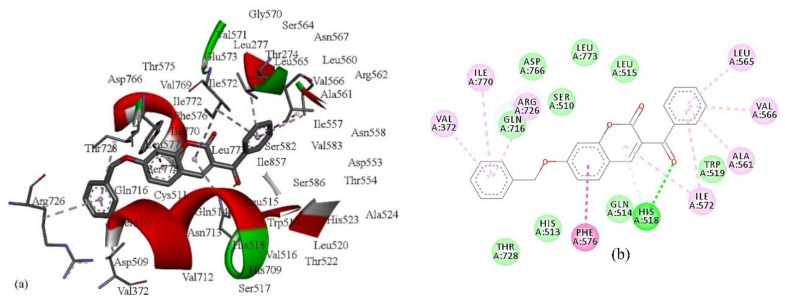
The main interactions of compound **7** with residues in the binding site of soybean lipoxygenase LOX-3: (**a**) 3D representation of the binding site; (**b**) 2D representation (green = conventional hydrogen bond; light green = *π*-donor hydrogen bond; purple = *π*–*π* T-shaped interactions; light purple = *π*–alkyl interactions).

**Figure 6:**
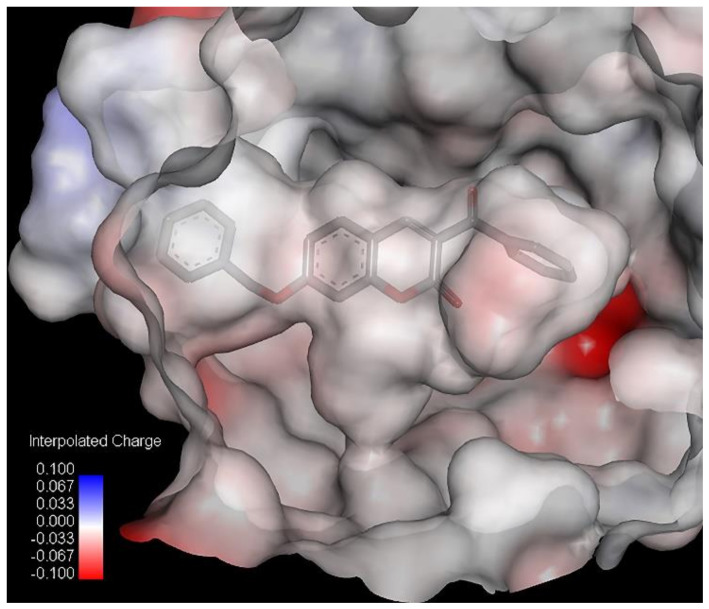
Charge surface representation of soybean LOX-3 binding site with docked compound **7**.

The molecular docking of analyzed compounds was performed on soybean lipoxygenase, soybean LOX-3. For this purpose, we have used the structure of soybean LOX-3 in complex with (−)-epigallocatechin gallate ((−)-EGCG) (PDB ID: 1JNQ) [45]. The docking scores of the docked poses with total energy < 90 kcal mol^−1^ are presented in Table 6. The docking scores of all the compounds, including the standard ligand, (−)-EGCG, are presented in Supplementary file 3 (Table S3). As in the case of molecular docking on 5-LOX, compound **7** obtained the best docking score. The total energy of binding is −128.06, most of which belongs to the energy of the van der Waals interaction (−118.67). The second-ranked compound is **6**, with the total energy −110.35 kcal mol^−1^, of which −97.09 kcal mol^−1^ belongs to the van der Waals interactions. Compound **6** had more binding energy through the H bond (−13.26 kcal mol^−1^) than compound **7**. The standard ligand, (−)-EGCG, achieved a lower docking score than eight coumarin derivatives with a total energy of −91.10 kcal mol^−1^, but interestingly, it obtained higher energy released by H-bond interactions than the other compounds (−20.32 kcal mol^−1^). Comparing the docking scores of the coumarin derivatives in docking with soybean LOX-3 (Table 6) with 5-LOX, it could be observed that, with the most active compound **7**, good binding affinity for both enzymes were shown for compounds **6**, **13**, **23***,* **and 35**. The main interactions of compound **7** with residues in the binding site of soybean LOX-3 are presented in Table 7. Interactions with all other compounds, including the standard ligand (−)-EGCG, are presented in the Supplementary file 3 (Table S3). Figure 5 illustrates the interactions of ligand 7 with the receptor 3-lipoxygenase in the binding site defined by the inhibitor (−)-EGCG. A charge surface representation of soybean LOX-3’s binding site with docked compound **7** is presented in Figure 6. 

The binding site of lipoxygenase-3 is a hydrophobic channel lined by the side chains of LEU273, THR274, LEU277, LEU560, ILE557, ARG755, ASP766, and ILE772 and where ASP766 participates in the hydrogen bonding network, as we confirmed in this study [46]. The docking results showed that most of the coumarin derivatives formed H bond interactions with the same amino acid residues—HIS513, GLN514, HIS518, TRP519, and ASP766—while the standard ligand, (−)-EGCG, makes one strong H bond interaction with HIS523. The oxygen atom from the benzoyl group of compound **7** creates a close H bond with HIS518 at a distance of 2.59 Å. The same atom creates H bonds with SER608 of 5-LOX. The benzene ring from chromene creates a strong π–π T-shaped interaction with the benzene ring of PHE576 (−8.21 kcal mol^−1^; 4.4 Å). The benzene ring from the benzyloxy group creates three *π*–alkyl interactions with ARG726 (4.24 Å), VAL372 (4.53 Å), and ILE770 (5.47 Å), as the benzene ring from the benzoyl group generates the same kind of van der Waals interactions with LEU 565 (4.30 Å), VAL566 (4.5 Å), ALA561 (4.62 Å), and ILE572 (4.83 Å). The pyran ring from the chromene formed a π-donor hydrogen bond with HIS518 (4.08 Å) and ILE572 (5.25 Å). The other compounds create van der Waals interactions with the same residues including the (−)-EGCG (Table S3). 

A docking study of both enzymes, 5-LOX and soybean LOX-3s revealed key structural features important for enhanced inhibitory activity, which are in agreement with the QSAR findings: the presence of an oxygen atom from the benzoyl group, which is important for the formation of hydrogen bonds, and a benzene ring for the generation of van der Waals interactions with amino acid residues in the binding sites of the enzymes.

In the study of Jothi et al. [47], the ligands epicatechin and gallic acid were docked deeply within the binding pocket region of soybean LOX-3, forming H-bond interactions with same residues—ASP766, GLN716, GLN514 and GLN716, and HIS518—the same as for the coumarin derivatives in the present study. Thiazolyl derivatives of mycophenolic acid were placed at the active site of the soybean LOX-3, interacting with the amino acids HIS518, LEU773, GLN716, and ASN713. The compound with the highest binding affinity formed hydrogen bonds with HIS518, ASN713, and GLN716 [48]. Similarly, as in the present study, the best docking scores for soybean LOX-3 (PDB ID: 1JNQ) and 5-LOX (PDB ID: 3V99) were obtained for the same compound that showed the best soybean lipoxygenase inhibition.

## 3. Materials and Methods 

### 3.1. Chemicals

1,1-diphenyl-picrylhydrazyl (DPPH), Trolox, nordihydroguaiaretic acid (NDGA), linoleic acid sodium salt, and lipoxidase Type I-B from Glycine max (soybean) were purchased from Sigma Chemical Co. (St. Louis, MO, USA); sodium phosphate monobasic monohydrate and sodium phosphate dibasic dehydrate were purchased from Merck KgaA, Merck Group (Darmstadt, Germany); boric acid, sodium tetraborate decahydrate, and tri-sodium phosphate hexahydrate were purchased from Honeywell Fluka^™^ (Charlotte, NC, USA); ammonium heptamolybdate tetrahydrate was purchased from Kemika d.d. (Zagreb, Croatia); dimethyl sulfoxide and sulfuric acid were purchased from Gram-Mol d.o.o. (Zagreb, Croatia); and 2,2′-azobis(2-methylpropionamidine) dihydrochloride and coumarin were purchased from Acros Organics B.V.B.A. (Geel, Belgium).

The coumarin derivatives (**1**–**39**) were synthesized and characterized as previously reported [28].

### 3.2. Soybean Lipoxygenase Activity Assay

Lipoxygenase activity was determined with linoleic acid sodium salt as a substrate. The reaction mixture (1 mL) contained 2 mM linoleic acid sodium salt and 150 units of soybean lipoxygenase in 0.2 M borate buffer (pH = 9). The reaction was carried out at 25 °C, and the increase in absorbance at 234 nm was measured over 100 s using a spectrophotometer, ThermoSpectronic, Helios Gamma (Thermo Fisher Scientific, Waltham, MA, USA). One unit of lipoxygenase activity was defined as that causing a change in the absorbance of 0.001 per min and mL of an enzyme. Activity measurements were carried out in triplicate.

### 3.3. Soybean Lipoxygenase Inhibition Assay 

Soybean lipoxygenase activity was measured in the presence of coumarin derivatives (Table 1). The compounds were dissolved in DMSO at 10 mM concentrations and added (10 µL) to the reaction mixture containing 840 µL of borate buffer (0.2M, pH = 9) and 100 µL of an aqueous solution of lipoxygenase (1500 U/mL) and pre-incubated for 5 min at 25 °C. The reaction was started by the addition of 50 µL of an aqueous solution of linoleic acid sodium salt (2 mM). Increases in the absorbance were monitored for 100 s at 234 nm. One unit of lipoxygenase activity was defined as the change in absorbance of 0.001 per min and mL of an enzyme. Activity measurements were carried out in triplicate. Controls without inhibitors but containing 10 μL of DMSO were routinely carried out.

The percent of inhibition of lipoxygenase-catalyzed reactions was calculated as follows:

Inhibition rate (%) = [1 − [(A_S_ – A_B_)/(A_C_ − A_B_)]] × 100 (3)

where A_S_ and A_B_ are the absorbances for the sample and blank, respectively, and A_C_ is the absorbance for the control without inhibitor but containing 10 μL of DMSO.

For the compounds showing significant inhibition of lipoxygenase activity at 100 µM concentrations, the IC_50_ value (a concentration giving a 50% inhibition of lipoxygenase activity) was determined. The IC_50_ values were calculated by plotting the inhibitor concentration (µM) against the percentage of lipoxygenase inhibition. The mathematical model used for that purpose was the “dose–response curve” ([Inhibitor] vs. normalized response—Variable slope) (GraphPad, 2020). The GraphPad software (GraphPad Prism version 8.4.2 (679), San Diego, CA, USA, www.graphpad.com) was used for the analysis. 

### 3.4. Inhibition of Linoleic Acid Lipid Peroxidation

Lipid peroxidation was determined as described previously [31]. The oxidation of linoleic acid in an aqueous dispersion results in the formation of conjugated diene hydroperoxide. AAPH (2,2′-azobis (2-methylpropionamidine) dihydrochloride) is used as a free radical initiator, and the reaction is monitored at 234 nm. To a UV cuvette, 930 µL of 0.05 M phosphate buffer (pH 7.4, pre-incubated at 37 °C), 10 µL of the 16 mM linoleic acid sodium salt solution, and 10 µL of coumarin dissolved in dimethyl sulfoxide (10 mM) were added. At the end, 50 µL of 40 mM AAPH solution was added. Lipid oxidation was measured in the presence of the same amount of DMSO. The results were compared with those for the appropriate standard inhibitor, Trolox. 

### 3.5. Determination of Reducing Activity of the Stable Radical 1,1-Diphenyl-Picrylhydrazyl (DPPH)

Antioxidant activity was determined according to Molnar et al. [49]. Briefly, all the compounds were prepared using DMSO as a solvent. Then, 750 µL of coumarin derivative solution (0.5 mM) and 750 µL of 0.2 mM DPPH radical solution were mixed and incubated for 30 min in the dark. The absorbance of each mixture was measured at 517 nm. The results were compared with those for the appropriate standards, NDGA and Trolox. 

### 3.6. Computational Methods

#### 3.6.1. QSAR Methods

The 3D structures of the 37 molecules were optimized by applying Avogadro 1.2.0 (University of Pittsburgh, Pittsburgh, PA, USA) using the molecular mechanics force field (MM+) [50]. Subsequently, all the structures were subjected to geometry optimization using the semiempirical PM3 method [51]. The molecular structures were optimized using the Polak–Ribiere algorithm until the root-mean-square gradient (RMS) was 0.001 kcal/(Åmol). Descriptor calculation was performed by using Parameter Client (Virtual Computational Chemistry Laboratory, an electronic remote version of the Dragon program) [52].

The elimination of irrelevant descriptors was performed using the Feature Selection command of ADMEWORKS ModelBuilder (Version 7.9.1.02011 Fujitsu Kyushu Systems Limited), Krakow, Poland, which includes the following tests: (a) the missing values test—which excludes descriptors with missing values; (b) the zero test—which excludes descriptors with less than the specified percentage of non-zero values; and (c) the automated correlations test—which deletes all parameters that have single or multiple correlations to other parameters, with *R*^2^ values larger than the specified threshold (0.7). The compounds for the test set were chosen with the aim of the Joining (Tree Clustering) method based on the whole set of descriptors, including the activity (log % inh.). As the distance measure, we used the Euclidean distance with the Single linkage as a linkage rule. The cluster analysis was performed by using Statistica 7.0 (StatSoft, Inc.; Tulsa, OK, USA).

The best QSAR models were obtained with a genetic algorithm (GA) using QSARINS (University of Insubria, Varese, Italy) [53]. The number of descriptors (*I*) in the multiple regression equation was limited to three. The models were assessed by fitting criteria, internal cross-validation using the leave-one out (LOO) method and Y-scrambling, and external validation. The fitting criteria included the coefficient of determination (*R*^2^); adjusted *R*^2^ (*R*^2^_adj_); cross-validated *R*^2^ using the leave-one-out method (*Q*^2^_LOO_); global correlation among the descriptors (*Kxx*); difference between the global correlations between the molecular descriptors and *y*, the response variable; global correlation among the descriptors (Δ*K*); standard deviation of regression (*s*); and Fisher ratio (*F*). Internal and external validations also included the following parameters: the coefficient of determination of the test set (*R*^2^*_ex_*), root-mean-square error of the training set (*RMSE_tr_*), root-mean-square error of the training set determined through the cross-validated LOO method (*RMSE_cv_*), root-mean-square error of the external validation set (*RMSE_ex_*), concordance correlation coefficient of the training set (*CCC_tr_*), test set using LOO cross-validation (*CCC_cv_*) and of the external validation set (*CCC_ex_*), mean absolute error of the training set (*MAE_tr_*), mean absolute error of the internal validation set (*MAE_cv_*), and mean absolute error of the external validation set (*MAE_ex_*). The analyzed external validation parameters also included the following. The statistical parameters Q^2^-F1, Q^2^-F2, Q^2^-F3, and *r^2^_m_* reflect the factual performance of the model regarding the true external predictivity of a QSAR model. The robustness of the QSAR models was tested by a Y-randomization test [37,38,54]. 

The investigation of the applicability domain of the prediction models was performed by leverage plotting (plotting residuals against the leverage of training compounds). The warning leverage h* is defined as 3*p*’/*n*, where *n* is the number of training compounds and *p*’ is the number of model adjustable parameters [55]. Tools of regression diagnostics such as residual plots and Williams plots were used to check the quality of the best models and define their applicability domain using QSARINS.

#### 3.6.2. Docking Studies

The molecular docking of compounds **1**–**35** was performed using iGEMDOCK (BioXGEM, Taiwan). The crystal coordinates of the 5-LOX in complex with arachidonic acid (PDB ID: 3V99) and soybean LOX-3 in complex with (−)-epigallocatechin gallate (PDB ID: 1JNQ) were downloaded from the Protein Data Bank (PDB, https://www.rcsb.org/) [24]. The 5-LOX and soybean LOX-3 structures were prepared, including the removal of water molecules and optimized the protein structure using BIOVIA Discovery Studio 4.5 (Dassault Systèmes, San Diego, CA, USA). Applying the generic evolutionary method, each compound was docked into the binding site (radius, 10 Å) of 5-LOX using the following parameters: the population size for 400 generations was 100, and the number of poses was 3. The protein–compound interaction profiles of the electrostatic (Elec), hydrogen-bonding (H bond), and van der Waals (vdW) interactions were also generated. The compounds were ranked by combining the pharmacological interactions and energy-based scoring function. The empirical scoring function is the total energy (kcal mol^−1^) of a predicted pose in the binding site and is estimated as follows: Total Energy = vdW + Hbond + Elec [56].

## 4. Conclusions

This study showed that some of the synthesized coumarin derivatives exhibited a significant inhibitory effect against soybean lipoxygenase. The coumarin with the highest inhibitory activity was 3-benzoyl-7-(benzyloxy)-2*H*-chromen-2-one (96.6%), followed by methyl 6-bromo-2-oxo-2*H*-chromene-3-carboxylate (85.1%) and 6-bromo-2-oxo-2*H*-chromene-3-carbonitrile (84.8%), both having bromine in position 6 of the coumarin ring. The QSAR analysis of the coumarin derivatives revealed the importance of the following characteristics for lipoxygenase inhibition: the presence of oxygen atoms from the benzoyl group, more atoms with higher polarizabilities in the area close to the geometrical center of the molecule, and the absence of pairs of these atoms at the topological distance 2. A molecular docking study confirmed the findings of the QSAR study regarding the structural features related to the inhibition of 5-LOX and soybean LOX-3, and indicated the importance of the 3-benzoyl ring as a substituent for the formation of hydrogen bonds and van der Waals interactions with binding site residues. 

## Figures and Tables

**Table 1:** Structures of analyzed compounds, values of experimentally determined inhibition of soybean lipoxygenase, inhibition of lipid peroxidation induced by AAPH and DPPH radical scavenging ability (at 100 µM concentrations of the compounds), and calculated logarithmic values of the % inhibition of lipoxygenase.

	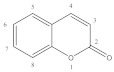							
No. mol	Mol ID	Substituents	DPPH (%)	LP inh. (%) (100 µM)	LOX inh. % (100 µM)	LOX inh. IC_50_ (µM)	log % LOX inh.	log % LOX inh. (calc. by eq) *
**1**	A1	3-acetyl	16.3	23.0	18.5	-	1.27	1.11
**2**	A2	3-methoxycarbonyl	32.1	16.6	7.1	-	0.85	1.04
**3**	A3	3- ethoxycarbonyl	33.5	7.0	14.3	-	1.15	1.09
**4**	A4	3-cyano	36.4	NA	14.8	-	1.17	1.37
**5**	A5	3-benzoyl	31.1	42.1	22.5	-	1.35	1.31
**6**	C4	3-cyano; 7-benzoyl	32.1	57.9	33.9	-	1.53	1.73
**7**	C5	3-benzoyl; 7-benzyloxy	36.3	86.1	96.6	26.82	1.98	1.97
**8**	COUM		33.2	2.6	23.1	-	1.36	1.31
**9**	D1	3-acetyl; 8-hydroxy	25.2	38.0	29.3	-	1.47	1.49
**10**	D4	3-cyano; 8-hydroxy	39.2	12.9	39.5	-	1.60	1.74
**11**	E1	3-acetyl; 7-hydroxy	15.9	29.8	45.1	-	1.65	1.57
**12**	E2	3-methoxycarbonyl; 7-hydroxy	15.2	44.7	37.4	-	1.57	1.51
**13**	E5	3-benzoyl; 7-hydroxy	32.7	55.2	76.1	13.98	1.88	1.79
**14**	F1	3-acetyl; 7-diethylamino	16.4	41.6	16.8	-	1.22	EXC.
**15**	G1	3-acetyl; 6-bromo	21.2	70.6	11.3	-	1.05	1.18
**16**	G2	3- methoxycarbonyl; 6-bromo	28.4	86.6	85.1	61.00	1.93	1.40
**17**	G3	3-ethoxycarbonyl; 6-bromo	32.9	76.7	55.2	97.48	1.74	1.45
**18**	G4	3-cyano; 6-bromo	48.4	81.0	84.8	64.77	1.93	1.77
**19**	J1	3-acetyl; 6-hydroxy	22.5	69.2	48.5	-	1.69	1.53
**20**	J2	3-methoxycarbonyl; 6-hydroxy	24.0	62.8	25.4	-	1.40	1.49
**21**	J3	3-ethoxycarbonyl; 6-hydroxy	58.1	47.1	51.9	17.40	1.71	1.54
**22**	J4	3-cyano; 6-hydroxy	39.0	66.8	29.3	-	1.47	1.77
**23**	J5	3-benzoyl; 6-hydroxy	38.9	66.5	55.4	88.55	1.74	1.77
**24**	K3	3-ethoxycarbonyl; 6-chloro	31.2	64.4	47.6	-	1.68	1.47
**25**	K5	3-benzoyl; 6-chloro	32.1	58.1	37.0	-	1.57	1.69
**26**	L3	3- ethoxycarbonyl; 6,8-dibromo	31.7	31.1	56.1	84.35	1.75	1.81
**27**	M2	3-methoxycarbonyl; 7-methoxy	21.0	61.9	25.2	-	1.40	1.56
**28**	M3	3-ethoxycarbonyl; 7-methoxy	32.9	91.0	36.5	-	1.56	1.59
**29**	M4	3-cyano; 7-methoxy	35.0	57.5	34.8	-	1.54	1.62
**30**	N2	3-methoxycarbonyl; 6-methoxy	20.5	75.3	76.0	52.54	1.88	1.53
**31**	N4	3-cyano; 6-methoxy	14.9	84.4	37.8	-	1.58	1.58
**32**	O1	3-acetyl; 8-ethoxy	19.6	36.1	18.0	-	1.26	1.55
**33**	O3	3-ethoxycarbonyl; 8-ethoxy	36.0	19.1	21.9	-	1.34	1.53
**34**	O4	3-cyano; 8-ethoxy	16.3	16.9	52.9	96.42	1.72	1.56
**35**	O5	3-benzoyl; 8-ethoxy	29.7	66.3	26.1	-	1.42	1.70
**36**	P2	3-methoxycarbonyl; 6-dihydroxyamino	22.9	29.7	40.5	-	1.61	1.53
**37**	P3	3-ethoxycarbonyl; 6-dihydroxyamino	33.1	29.1	46.6	-	1.67	1.61
**38**	L5	3-benzoyl; 6,8-dibromo	35.7	73.0	-	-	-	-
**39**	M5	3-benzoyl; 7-methoxy	29.3	69.2	-	-	-	-
**40**	Trolox	-	77.0	61.8	-	-	-	-
**41**	NDGA	-	56.5	NT	-	NT	-	-

NA, no activity; NT, not tested; NDGA, nordihydroguaiaretic acid; DPPH-1, 1-diphenyl-picrylhydrazyl; LP, lipid peroxidation; LOX inh., soybean lipoxygenase inhibition; EXC., excluded as outlier from training set. * Calculated by quantitative structure-activity relationship (QSAR) equation: log% inhibition = 0.67 + 0.42 (0.72) *C-026* + 0.07 (0.43) *RDF035p*-1.85 (−0.36) *HATS8p.*

**Table 2:** The statistical results of QSAR models (1)–(2).

Statistical Parameters	Model 1	Model 2
*N*_tr_	29	28
*N*_ex_	8	8
*R* ^2^	0.56	0.68
*R* ^2^_adj_	0.50	0.64
*s*	0.2	0.17
*F*	10.44	17.27
*Kxx*	0.14	0.14
Δ*K*	0.16	0.18
*RMSE_tr_*	0.96	0.15
*MAE_tr_*	0.15	0.13
*CCC_tr_*	0.71	0.81
*Q^2^_LOO_*	0.41	0.60
*RMSE_cv_*	0.21	0.18
*MAE_cv_*	0.17	0.15
*CCC_cv_*	0.61	0.75
*R^2^_Y_scr*	0.11	0.11
*Q^2^_Y_scr*	−0.23	−0.23
*RMSE_ext_*	0.25	0.26
*MAE_ext_*	0.20	0.21
*R^2^_ext_*	0.80	0.80
*CCC_ext_*	−0.76	−0.85
*Q^2^*_F*1*_	−0.71	−1.08
*Q^2^*_F*2*_	−1.71	−2.10
*Q^2^*_F*3*_	0.20	0.08
*r^2^_m_* average	−0.77	−0.72
*r^2^_m_* difference	0.64	0.33
Applicability domain		
*N* compounds outlier	1 (**14**)	1 (**16**)
*N* compounds out of app.dom.	-	1 (**15**)

LOO (leave-one out); *R*^2^ (coefficient of determination); *R*^2^_adj_ (adjusted coefficient of determination); *s* (standard deviation of regression); *F* (Fisher ratio); *Kxx* (global correlation among descriptors); Δ*K* (global correlation among descriptors); *RMSE_tr_* (root-mean-square error of the training set); *MAE_tr_* (mean absolute error of the training set); *CCC_tr_* (concordance correlation coefficient of the training set); *Q^2^_LOO_* (cross-validated explained variance); *RMSE_cv_* (root-mean-square error of the training set determined through the cross validated method); *MAE_cv_* (mean absolute error of the internal validation set); *CCC_cv_* (concordance correlation coefficient test set using cross validation); *R*^2^*_Y_scr* (Y-scramble correlation coefficients); *Q*^2^*_Yscr_* (Y-scramble cross-validation coefficients); *RMSE_ex_* (root-mean-square error of the external validation set); *MAE_ex_* (mean absolute error of the external validation set); *R*^2^*_ext_* (coefficient of determination of validation set); *Q*^2^*_F_*_1_, *Q*^2^*_F_*_2_, *Q*^2^*_F_*_3_ (predictive squared correlation coefficients); *CCC_ext_* (concordance correlation coefficient of the test set); *r^2^_m_* average (average value of squared correlation coefficients between the observed and leave-one-out predicted values of the compounds with and without intercept); *r^2^_m_* difference (absolute difference between the observed and leave-one-out predicted values of the compounds with and without intercept); h* (warning leverage for the applicability domain of the model).

**Table 3:** Correlation matrix (correlation coefficient, R) for the descriptors included in models (1)–(2).

	*C-026*	*HATS8p*	*RDF035p*
*C-026*	1		
*HATS8p*	0.25	1.00	
*RDF035p*	0.00	−0.03	1.00

**Table 4:** Docking score energies of the interactions of the best-docked poses of the coumarin derivatives in complex with 5-lipoxygenase.

Compound (Pose)	Total Energy/kcal mol^−1^	Van der Waals Interaction/kcal mol^−1^	H Bond/kcal mol^−1^
**7** (2)	−126.2	−118.2	−8.0
**Arachidonic acid**	−120.58	−120.58	0
**35** (2)	−116.2	−109.2	−7.0
**23** (2)	−108.7	−99.6	−9.1
**33** (2)	−107.8	−99.3	−8.5
**13** (0)	−105.3	−91.1	−14.2
**28** (1)	−105.1	−91.7	−13.4
**6** (0)	−103.8	−93.3	−10.5
**5** (1)	−103.5	−96.5	−7.0
**24** (0)	−102.1	−88.4	−13.7
**25** (2)	−102.0	−95.0	−7.0
**21** (1)	−101.8	−88.2	−13.6
**17** (0)	−101.7	−88.0	−13.7
**26** (0)	−100.4	−94.9	−5.4

**Table 5:** The energies of the main interactions between 5-lipoxygenase residues and compound **7**.

H Bond	Energy	Van der Waals Interaction	Energy
H-S-ASN-554	−3.27	V-S-PHE-610	−14.93
H-S-SER-608	−2.5	V-S-PHE-555	−11.18
H-S-GLN-557	−2.2	V-M-PHE-555	−11.02
		V-M-TYR-558	−10.86
		V-M-LEU-607	−10.09
		V-M-SER-608	−9.50
		V-S-TYR-558	−7.45
		V-S-LEU-607	−7.32
		V-S-GLN-557	−6.12
		V-M-GLN-557	−5.52
		V-M-ASN-554	−4.79
		V-S-SER-608	−4.74
		V-S-ASN-554	−4.17
		V-M-VAL-604	−2.77
		V-M-ALA-672	−1.25
		V-S-VAL-604	−1.15

(M = main chain; S = side chain).

**Table 6:** Docking score energies of the interactions of the best-docked poses of coumarin derivatives, including the standard ligand (−)-EGCG * in complex with lipoxygenase-3.

Compound (Pose)	Total Energy/kcal mol^−1^	Van der Waals Interaction/kcal mol^−1^	H Bond/kcal mol^−1^
**7** (0)	−128.06	−118.67	−9.39
**6** (2)	−110.35	−97.09	−13.26
**14** (2)	−99.53	−86.94	−12.59
**13** (0)	−97.95	−82.23	−15.72
**23** (2)	−97.32	−84.60	−12.72
**35** (0)	−96.18	−76.96	−19.22
**37** (1)	−95.74	−84.18	−11.56
**25** (2)	−93.46	−86.48	−6.98
**(−)-EGCG** (0)	−91.10	−70.79	−20.32
**29** (1)	−90.78	−80.30	−10.48
**36** (2)	−90.26	−70.50	−19.76

* (−)-epigallocatechin gallate.

**Table 7:** The energies of the main interactions between lipoxygenase-3 residues and compound **7**.

H Bond	Energy/kcal mol^−1^	Van der Waals Interaction	Energy/kcal mol^−1^
S-HIS-518	−3.40	M-SER-510	−3.80
M-TRP-519	−3.50	S-HIS-513	−6.56
S-ASP-766	−2.49	M-GLN-514	−2.41
		S-GLN-514	−3.78
		S-HIS-518	−7.57
		S-TRP-519	−9.61
		S-HIS-523	−1.46
		S-LEU-565	−2.53
		S-ILE-572	−7.90
		S-PHE-576	−8.21
		S-GLN-716	−10.52
		S-ARG-726	−7.33
		S-ASP-766	−3.78
		S-ILE-770	−3.75
		S-LEU-773	−2.83

## Supplementary Materials

The following are available online at https://www.mdpi.com/1424-8247/13/7/154/s1: Table S1: The values of the descriptors included in model (2); Table S2: The docking scores and main interaction energies of the best docking poses of tested coumarin derivatives in complex with 5-LOX; Table S3: The docking scores and main interaction energies of the best docking poses of tested coumarin derivatives in complex with 3-LOX.
